# Aqua­(dicyanamido)­{μ-6,6′-dimeth­oxy-2,2′-[ethane-1,2-diylbis(nitrilo­methyl­i­dyne)]diphenolato}copper(II)sodium(I)

**DOI:** 10.1107/S1600536809011064

**Published:** 2009-03-31

**Authors:** Yong-Miao Shen, Wei Wang

**Affiliations:** a Department of Chemistry, Shaoxing University, Shaoxing 312000, People’s Republic of China; b Yancheng Institute of Technology, School of Chemical and Biological Engineering, Yancheng 224003, People’s Republic of China

## Abstract

The mol­ecule of the title compound, [CuNa(C_18_H_18_N_2_O_4_)(C_2_N_3_)(H_2_O)], is almost planar, the maximum deviation from the mol­ecular plane being 0.48 (4) Å. The coordination environment of the Cu^2+^ ion is distorted square-planar and it is *N*_2_ *O*_2_-chelated by the Schiff base ligand. The Na^+^ cation has a distorted octahedral environment defined by the four O atoms of the 6,6′-dimeth­oxy-2,2′-[ethane-1,2-diylbis(nitrilo­methyl­idyne)]diphenolate ligand, a water ligand and a dicyanamide anion.

## Related literature

For chemical background, see: Ohba & Okawa (2000 ▶). For related structures, see: Correia *et al.* (2005 ▶); Costes *et al.*(2004 ▶).
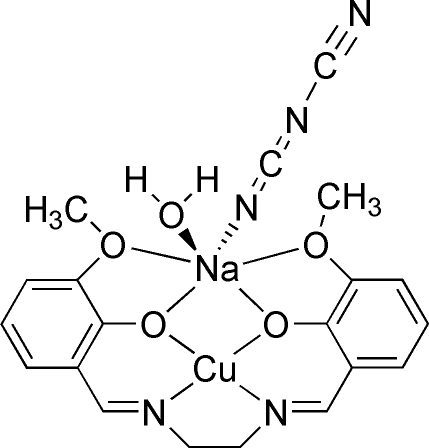


## Experimental

### 

#### Crystal data

[CuNa(C_18_H_18_N_2_O_4_)(C_2_N_3_)(H_2_O)]
*M* *_r_* = 496.94
Monoclinic, 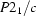

*a* = 7.5974 (14) Å
*b* = 22.999 (4) Å
*c* = 12.876 (3) Å
β = 101.986 (4)°
*V* = 2200.7 (7) Å^3^
*Z* = 4
Mo *K*α radiation
μ = 1.05 mm^−1^
*T* = 293 K
0.23 × 0.21 × 0.19 mm

#### Data collection

Bruker APEXII CCD area-detector diffractometer
Absorption correction: multi-scan (*SADABS*; Sheldrick, 2003 ▶) *T*_min_ = 0.794, *T*_max_ = 0.825
11729 measured reflections
4314 independent reflections
2996 reflections with *I* > 2σ(*I*)
*R*_int_ = 0.034

#### Refinement

*R*[*F* ^2^ > 2σ(*F* ^2^)] = 0.049
*wR*(*F* ^2^) = 0.142
*S* = 1.03
4314 reflections
291 parameters
54 restraints
H-atom parameters constrained
Δρ_max_ = 0.41 e Å^−3^
Δρ_min_ = −0.49 e Å^−3^

### 

Data collection: *APEX2* (Bruker, 2004 ▶); cell refinement: *SAINT-Plus* (Bruker, 2001 ▶); data reduction: *SAINT-Plus*; program(s) used to solve structure: *SHELXS97* (Sheldrick, 2008 ▶); program(s) used to refine structure: *SHELXL97* (Sheldrick, 2008 ▶); molecular graphics: *SHELXTL* (Sheldrick, 2008 ▶); software used to prepare material for publication: *SHELXTL*.

## Supplementary Material

Crystal structure: contains datablocks I, global. DOI: 10.1107/S1600536809011064/hg2492sup1.cif

Structure factors: contains datablocks I. DOI: 10.1107/S1600536809011064/hg2492Isup2.hkl

Additional supplementary materials:  crystallographic information; 3D view; checkCIF report

## Figures and Tables

**Table 1:** Hydrogen-bond geometry (Å, °)

*D*—H⋯*A*	*D*—H	H⋯*A*	*D*⋯*A*	*D*—H⋯*A*
O5—H5*B*⋯N5^i^	0.81	2.02	2.826 (5)	178
O5—H5*A*⋯N3^ii^	0.81	2.15	2.961 (5)	173

Symmetry codes: (i) 
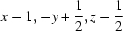
; (ii) 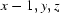.

**Fig. 1.**
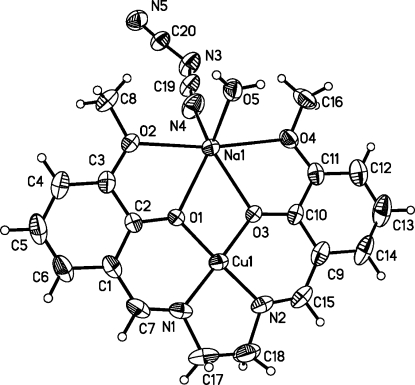
The independent molecules of the title compound, showing 30% probability displacement ellipsoids and the atom-numbering scheme.

## supplementary crystallographic information

### Comment

The dicyanamide ligand N(CN)_2_, has attracted attention in the past four years for the buildup of interesting extended architectures. Its versatile coordination behavior and its ability to organize solids into polymeric structures with a rich diversity of magnetic properties have attracted interest toward this research area (Ohba *et al.*, 2000). *N*,*N*-disalicylideneethylenediamine type Schiff bases ligands present versatile steric, electronic and lipophilic properties (Correia *et al.* 2005). We report here the synthesis and crystal structure of the title compound.

The molecular structure is shown in Fig.1. The values of the geometric parameters in (I) are normal (Costes *et al.* 2004) (Table 1). The copper and sodium cations are connected *via* two bridging oxygen atoms of the ligand. The Na atom is coordinated by the four O atoms of the 6,6'-Dimethoxy-2,2'-(ethane-1,2-diyldiiminodimethylene)diphenol ligand, a water ligand and a dicyanamide anion while the four-coordinate Cu gives a planar coordination.

### Experimental

A mixture of 6,6'-Dimethoxy-2,2'-(ethane-1,2-diyldiiminodimethylene)diphenol (1 mmol) and copper chloride (1 mmol) in methanol (15 ml) was stirred for 30 min and sodium dicyanamide (1 mmol) was added, stirred for another 15 min and then filtered. The resulting clear blue solution was vapor at room temperature for 7 days, after which large blue block-shaped crystals of the title complex suitable for X-ray diffraction analysis were obtained.

### Refinement

The H atoms were fixed geometrically and were treated as riding on their parent C atoms, with C–H distances in the range of 0.93–0.97Å and with *U*_iso_(H) = 1.2*U*_eq_(parent atom), or *U*_iso_(H) = 1.5*U*_eq_(C_methyl_).

### Crystal data

**Table:**

[CuNa(C_18_H_18_N_2_O_4_)(C_2_N_3_)(H_2_O)]	*F*(000) = 1020
*M**_r_* = 496.94	*D*_x_ = 1.500 Mg m^−^^3^*D*_m_ = 1.500 Mg m^−^^3^*D*_m_ measured by not measured
Monoclinic, *P*2_1_/*c*	Mo *K*α radiation, λ = 0.71073 Å
Hall symbol: -P 2ybc	Cell parameters from 3562 reflections
*a* = 7.5974 (14) Å	θ = 2.8–25.0°
*b* = 22.999 (4) Å	µ = 1.05 mm^−^^1^
*c* = 12.876 (3) Å	*T* = 293 K
β = 101.986 (4)°	Block, blue
*V* = 2200.7 (7) Å^3^	0.23 × 0.21 × 0.19 mm
*Z* = 4	

### Data collection

**Table:**

Bruker APEXII CCD area-detector diffractometer	4314 independent reflections
Radiation source: fine-focus sealed tube	2996 reflections with *I* > 2σ(*I*)
graphite	*R*_int_ = 0.034
φ and ω scans	θ_max_ = 26.0°, θ_min_ = 1.8°
Absorption correction: multi-scan (*SADABS*; Sheldrick, 2003)	*h* = −9→8
*T*_min_ = 0.794, *T*_max_ = 0.825	*k* = −27→28
11729 measured reflections	*l* = −15→15

### Refinement

**Table:**

Refinement on *F*^2^	Primary atom site location: structure-invariant direct methods
Least-squares matrix: full	Secondary atom site location: difference Fourier map
*R*[*F*^2^ > 2σ(*F*^2^)] = 0.049	Hydrogen site location: inferred from neighbouring sites
*wR*(*F*^2^) = 0.142	H-atom parameters constrained
*S* = 1.03	*w* = 1/[σ^2^(*F*_o_^2^) + (0.0747*P*)^2^ + 0.4736*P*] where *P* = (*F*_o_^2^ + 2*F*_c_^2^)/3
4314 reflections	(Δ/σ)_max_ = 0.001
291 parameters	Δρ_max_ = 0.41 e Å^−^^3^
54 restraints	Δρ_min_ = −0.49 e Å^−^^3^

### Special details

**Table:**

Geometry. All e.s.d.'s (except the e.s.d. in the dihedral angle between two l.s. planes) are estimated using the full covariance matrix. The cell e.s.d.'s are taken into account individually in the estimation of e.s.d.'s in distances, angles and torsion angles; correlations between e.s.d.'s in cell parameters are only used when they are defined by crystal symmetry. An approximate (isotropic) treatment of cell e.s.d.'s is used for estimating e.s.d.'s involving l.s. planes.
Refinement. Refinement of *F*^2^ against ALL reflections. The weighted *R*-factor *wR* and goodness of fit *S* are based on *F*^2^, conventional *R*-factors *R* are based on *F*, with *F* set to zero for negative *F*^2^. The threshold expression of *F*^2^ > σ(*F*^2^) is used only for calculating *R*-factors(gt) *etc*. and is not relevant to the choice of reflections for refinement. *R*-factors based on *F*^2^ are statistically about twice as large as those based on *F*, and *R*- factors based on ALL data will be even larger.

### Fractional atomic coordinates and isotropic or equivalent isotropic displacement parameters (Å^2^)

**Table:**

	*x*	*y*	*z*	*U*_iso_*/*U*_eq_	
Cu1	0.24890 (6)	0.506744 (19)	0.06032 (4)	0.04808 (19)	
Na1	0.36641 (19)	0.38275 (6)	0.20334 (11)	0.0492 (4)	
O1	0.2721 (4)	0.48218 (10)	0.1952 (2)	0.0513 (6)	
O2	0.3153 (4)	0.42658 (12)	0.3689 (2)	0.0705 (8)	
O3	0.3315 (3)	0.43211 (10)	0.03987 (18)	0.0472 (6)	
O4	0.4284 (4)	0.32308 (11)	0.0497 (2)	0.0619 (7)	
O5	0.1622 (4)	0.30926 (12)	0.2164 (2)	0.0690 (8)	
H5B	0.1286	0.2876	0.1668	0.083*	
H5A	0.1030	0.3044	0.2614	0.083*	
N1	0.1661 (4)	0.58079 (13)	0.0845 (3)	0.0549 (8)	
N2	0.2233 (4)	0.52876 (15)	−0.0761 (3)	0.0556 (8)	
N3	0.9191 (5)	0.2881 (2)	0.3649 (3)	0.0874 (10)	
N4	0.6425 (6)	0.3426 (2)	0.2955 (3)	0.0896 (11)	
N5	1.0355 (6)	0.26675 (19)	0.5447 (3)	0.0868 (12)	
C1	0.1804 (5)	0.57156 (17)	0.2676 (4)	0.0603 (11)	
C2	0.2396 (5)	0.51238 (16)	0.2741 (3)	0.0492 (9)	
C3	0.2601 (6)	0.48344 (18)	0.3710 (3)	0.0592 (10)	
C4	0.2247 (7)	0.5129 (2)	0.4559 (4)	0.0818 (15)	
H4	0.2371	0.4938	0.5207	0.098*	
C5	0.1700 (8)	0.5713 (3)	0.4488 (5)	0.0953 (17)	
H5	0.1493	0.5903	0.5089	0.114*	
C6	0.1479 (7)	0.5995 (2)	0.3589 (4)	0.0807 (14)	
H6	0.1105	0.6381	0.3553	0.097*	
C7	0.1477 (6)	0.60141 (17)	0.1718 (4)	0.0656 (12)	
H7	0.1090	0.6398	0.1724	0.079*	
C8	0.3386 (8)	0.3935 (2)	0.4617 (3)	0.0885 (16)	
H8A	0.4210	0.4129	0.5175	0.133*	
H8B	0.3861	0.3560	0.4494	0.133*	
H8C	0.2248	0.3887	0.4820	0.133*	
C9	0.3045 (5)	0.4367 (2)	−0.1448 (3)	0.0630 (11)	
C10	0.3413 (5)	0.40704 (17)	−0.0485 (3)	0.0482 (9)	
C11	0.3903 (5)	0.34713 (18)	−0.0474 (3)	0.0575 (10)	
C12	0.4003 (7)	0.3185 (2)	−0.1381 (4)	0.0846 (15)	
H12	0.4310	0.2794	−0.1364	0.101*	
C13	0.3647 (9)	0.3482 (4)	−0.2317 (5)	0.112 (2)	
H13	0.3715	0.3289	−0.2943	0.134*	
C14	0.3192 (8)	0.4058 (3)	−0.2353 (4)	0.0973 (18)	
H14	0.2974	0.4249	−0.3004	0.117*	
C15	0.2507 (5)	0.4971 (2)	−0.1515 (3)	0.0637 (12)	
H15	0.2347	0.5145	−0.2181	0.076*	
C16	0.4985 (7)	0.26442 (18)	0.0605 (4)	0.0851 (15)	
H16A	0.4074	0.2379	0.0260	0.128*	
H16B	0.5331	0.2547	0.1345	0.128*	
H16C	0.6014	0.2618	0.0283	0.128*	
C17	0.1101 (7)	0.6168 (2)	−0.0103 (4)	0.0847 (15)	
H17A	−0.0201	0.6196	−0.0281	0.102*	
H17B	0.1590	0.6557	0.0029	0.102*	
C18	0.1742 (8)	0.5909 (2)	−0.0959 (4)	0.0906 (16)	
H18A	0.2789	0.6122	−0.1072	0.109*	
H18B	0.0819	0.5939	−0.1602	0.109*	
C19	0.7693 (7)	0.3174 (2)	0.3323 (3)	0.0738 (10)	
C20	0.9740 (6)	0.2781 (2)	0.4624 (4)	0.0659 (10)	

### Atomic displacement parameters (Å^2^)

**Table:**

	*U*^11^	*U*^22^	*U*^33^	*U*^12^	*U*^13^	*U*^23^
Cu1	0.0467 (3)	0.0467 (3)	0.0492 (3)	−0.0028 (2)	0.0064 (2)	0.0091 (2)
Na1	0.0568 (9)	0.0453 (8)	0.0444 (8)	0.0046 (7)	0.0079 (6)	0.0042 (6)
O1	0.0734 (18)	0.0380 (13)	0.0434 (14)	0.0046 (12)	0.0140 (12)	0.0000 (11)
O2	0.115 (3)	0.0577 (17)	0.0409 (15)	0.0092 (17)	0.0201 (15)	0.0046 (13)
O3	0.0575 (15)	0.0475 (14)	0.0362 (13)	0.0008 (12)	0.0090 (11)	0.0018 (11)
O4	0.0793 (19)	0.0461 (15)	0.0653 (19)	0.0003 (13)	0.0264 (15)	−0.0075 (13)
O5	0.088 (2)	0.0678 (18)	0.0566 (17)	−0.0200 (16)	0.0264 (15)	−0.0101 (14)
N1	0.0471 (18)	0.0396 (17)	0.074 (2)	−0.0016 (14)	0.0039 (16)	0.0128 (16)
N2	0.0464 (18)	0.063 (2)	0.053 (2)	−0.0063 (15)	0.0016 (15)	0.0218 (17)
N3	0.076 (2)	0.125 (2)	0.0618 (19)	0.0281 (19)	0.0156 (17)	0.0111 (19)
N4	0.077 (2)	0.121 (3)	0.065 (2)	0.025 (2)	0.0029 (17)	0.0089 (19)
N5	0.097 (3)	0.087 (3)	0.071 (2)	0.019 (2)	0.003 (2)	0.015 (2)
C1	0.056 (2)	0.050 (2)	0.072 (3)	0.0021 (19)	0.008 (2)	−0.019 (2)
C2	0.051 (2)	0.047 (2)	0.050 (2)	−0.0018 (17)	0.0113 (17)	−0.0081 (17)
C3	0.065 (3)	0.065 (3)	0.049 (2)	−0.002 (2)	0.0145 (19)	−0.011 (2)
C4	0.095 (4)	0.104 (4)	0.047 (3)	0.000 (3)	0.015 (2)	−0.017 (2)
C5	0.113 (4)	0.098 (4)	0.076 (4)	0.009 (4)	0.024 (3)	−0.043 (3)
C6	0.083 (3)	0.068 (3)	0.089 (4)	0.016 (3)	0.015 (3)	−0.031 (3)
C7	0.061 (3)	0.037 (2)	0.093 (4)	0.0035 (19)	0.003 (2)	−0.006 (2)
C8	0.126 (4)	0.097 (4)	0.046 (3)	0.000 (3)	0.026 (3)	0.019 (2)
C9	0.044 (2)	0.102 (4)	0.043 (2)	−0.003 (2)	0.0081 (17)	−0.002 (2)
C10	0.040 (2)	0.068 (2)	0.037 (2)	−0.0084 (18)	0.0090 (15)	−0.0053 (18)
C11	0.055 (2)	0.066 (3)	0.056 (3)	−0.010 (2)	0.0186 (19)	−0.019 (2)
C12	0.089 (4)	0.091 (4)	0.075 (3)	−0.002 (3)	0.022 (3)	−0.031 (3)
C13	0.119 (5)	0.160 (6)	0.059 (4)	0.019 (5)	0.024 (3)	−0.037 (4)
C14	0.099 (4)	0.158 (6)	0.036 (3)	0.007 (4)	0.017 (2)	−0.008 (3)
C15	0.048 (2)	0.101 (4)	0.040 (2)	−0.006 (2)	0.0053 (18)	0.019 (2)
C16	0.110 (4)	0.044 (2)	0.109 (4)	−0.001 (2)	0.041 (3)	−0.011 (2)
C17	0.079 (3)	0.074 (3)	0.099 (4)	0.016 (3)	0.014 (3)	0.046 (3)
C18	0.109 (4)	0.079 (3)	0.079 (4)	0.001 (3)	0.009 (3)	0.036 (3)
C19	0.069 (2)	0.103 (3)	0.0486 (19)	0.016 (2)	0.0115 (18)	0.0103 (19)
C20	0.062 (2)	0.082 (2)	0.0536 (19)	0.0124 (18)	0.0107 (17)	0.0120 (19)

### Geometric parameters (Å, °)

**Table:**

Cu1—O1	1.799 (2)	C3—C4	1.360 (6)
Cu1—N2	1.800 (3)	C4—C5	1.404 (7)
Cu1—N1	1.864 (3)	C4—H4	0.9300
Cu1—O3	1.865 (2)	C5—C6	1.306 (7)
Na1—O5	2.324 (3)	C5—H5	0.9300
Na1—O3	2.357 (3)	C6—H6	0.9300
Na1—N4	2.373 (4)	C7—H7	0.9300
Na1—O1	2.392 (3)	C8—H8A	0.9600
Na1—O2	2.461 (3)	C8—H8B	0.9600
Na1—O4	2.531 (3)	C8—H8C	0.9600
O1—C2	1.297 (4)	C9—C14	1.389 (7)
O2—C3	1.375 (5)	C9—C10	1.392 (5)
O2—C8	1.397 (5)	C9—C15	1.446 (6)
O3—C10	1.292 (4)	C10—C11	1.427 (6)
O4—C11	1.343 (5)	C11—C12	1.356 (6)
O4—C16	1.446 (5)	C12—C13	1.363 (8)
O5—H5B	0.8078	C12—H12	0.9300
O5—H5A	0.8118	C13—C14	1.368 (9)
N1—C7	1.254 (6)	C13—H13	0.9300
N1—C17	1.463 (5)	C14—H14	0.9300
N2—C15	1.264 (5)	C15—H15	0.9300
N2—C18	1.485 (6)	C16—H16A	0.9600
N3—C20	1.260 (5)	C16—H16B	0.9600
N3—C19	1.314 (6)	C16—H16C	0.9600
N4—C19	1.139 (5)	C17—C18	1.426 (7)
N5—C20	1.097 (5)	C17—H17A	0.9700
C1—C7	1.389 (6)	C17—H17B	0.9700
C1—C6	1.406 (6)	C18—H18A	0.9700
C1—C2	1.430 (5)	C18—H18B	0.9700
C2—C3	1.393 (6)		
			
O1—Cu1—N2	177.98 (13)	C6—C5—C4	121.0 (5)
O1—Cu1—N1	95.42 (13)	C6—C5—H5	119.5
N2—Cu1—N1	86.24 (16)	C4—C5—H5	119.5
O1—Cu1—O3	83.04 (10)	C5—C6—C1	120.1 (5)
N2—Cu1—O3	95.30 (14)	C5—C6—H6	120.0
N1—Cu1—O3	178.45 (13)	C1—C6—H6	120.0
O5—Na1—O3	117.51 (11)	N1—C7—C1	125.2 (4)
O5—Na1—N4	102.41 (15)	N1—C7—H7	117.4
O3—Na1—N4	123.91 (13)	C1—C7—H7	117.4
O5—Na1—O1	120.00 (11)	O2—C8—H8A	109.5
O3—Na1—O1	61.53 (9)	O2—C8—H8B	109.5
N4—Na1—O1	128.31 (15)	H8A—C8—H8B	109.5
O5—Na1—O2	90.42 (11)	O2—C8—H8C	109.5
O3—Na1—O2	124.57 (10)	H8A—C8—H8C	109.5
N4—Na1—O2	90.49 (13)	H8B—C8—H8C	109.5
O1—Na1—O2	63.05 (9)	C14—C9—C10	117.4 (5)
O5—Na1—O4	84.12 (10)	C14—C9—C15	120.8 (5)
O3—Na1—O4	64.40 (9)	C10—C9—C15	121.8 (4)
N4—Na1—O4	83.75 (13)	O3—C10—C9	121.9 (4)
O1—Na1—O4	125.91 (10)	O3—C10—C11	119.0 (3)
O2—Na1—O4	171.02 (11)	C9—C10—C11	119.1 (4)
C2—O1—Cu1	126.3 (2)	O4—C11—C12	124.2 (4)
C2—O1—Na1	125.6 (2)	O4—C11—C10	114.4 (3)
Cu1—O1—Na1	108.12 (11)	C12—C11—C10	121.4 (4)
C3—O2—C8	119.1 (3)	C11—C12—C13	118.9 (5)
C3—O2—Na1	120.7 (2)	C11—C12—H12	120.6
C8—O2—Na1	120.2 (3)	C13—C12—H12	120.6
C10—O3—Cu1	128.2 (2)	C12—C13—C14	121.2 (5)
C10—O3—Na1	123.7 (2)	C12—C13—H13	119.4
Cu1—O3—Na1	107.21 (11)	C14—C13—H13	119.4
C11—O4—C16	118.6 (3)	C13—C14—C9	122.1 (5)
C11—O4—Na1	117.7 (2)	C13—C14—H14	119.0
C16—O4—Na1	123.7 (3)	C9—C14—H14	119.0
Na1—O5—H5B	119.8	N2—C15—C9	126.6 (4)
Na1—O5—H5A	128.9	N2—C15—H15	116.7
H5B—O5—H5A	110.5	C9—C15—H15	116.7
C7—N1—C17	117.8 (4)	O4—C16—H16A	109.5
C7—N1—Cu1	126.8 (3)	O4—C16—H16B	109.5
C17—N1—Cu1	115.3 (3)	H16A—C16—H16B	109.5
C15—N2—C18	119.8 (4)	O4—C16—H16C	109.5
C15—N2—Cu1	125.9 (3)	H16A—C16—H16C	109.5
C18—N2—Cu1	114.2 (3)	H16B—C16—H16C	109.5
C20—N3—C19	119.8 (4)	C18—C17—N1	108.8 (4)
C19—N4—Na1	171.6 (5)	C18—C17—H17A	109.9
C7—C1—C6	119.1 (4)	N1—C17—H17A	109.9
C7—C1—C2	121.2 (4)	C18—C17—H17B	109.9
C6—C1—C2	119.6 (4)	N1—C17—H17B	109.9
O1—C2—C3	116.2 (3)	H17A—C17—H17B	108.3
O1—C2—C1	124.9 (4)	C17—C18—N2	112.5 (4)
C3—C2—C1	118.8 (4)	C17—C18—H18A	109.1
C4—C3—O2	126.9 (4)	N2—C18—H18A	109.1
C4—C3—C2	118.5 (4)	C17—C18—H18B	109.1
O2—C3—C2	114.5 (3)	N2—C18—H18B	109.1
C3—C4—C5	121.9 (5)	H18A—C18—H18B	107.8
C3—C4—H4	119.0	N4—C19—N3	174.1 (5)
C5—C4—H4	119.0	N5—C20—N3	173.2 (5)

### Hydrogen-bond geometry (Å, °)

**Table:**

*D*—H···*A*	*D*—H	H···*A*	*D*···*A*	*D*—H···*A*
O5—H5B···N5^i^	0.81	2.02	2.826 (5)	178
O5—H5A···N3^ii^	0.81	2.15	2.961 (5)	173

Symmetry codes: (i) *x*−1, −*y*+1/2, *z*−1/2; (ii) *x*−1, *y*, *z*.
